# Blood biomarkers associated with acute type II respiratory failure in COPD: A meta‐analysis

**DOI:** 10.1111/crj.13464

**Published:** 2022-01-10

**Authors:** Tieying Shi, Li Feng

**Affiliations:** ^1^ Department of Geriatrics, Zhejiang Provincial People's Hospital People's Hospital of Hangzhou Medical College Hangzhou China

**Keywords:** chronic obstructive pulmonary disease, meta‐analysis, risk factors, type II respiratory failure

## Abstract

**Objective:**

This study aims to summarize the risk factors of type II respiratory failure in patients with an acute exacerbation of chronic obstructive pulmonary disease (COPD), to guide clinical treatment in time, and consequently reduce the serious impact of COPD on human health.

**Methods:**

Five databases were searched for relevant articles on risk factors of acute exacerbation of COPD combinate with type II respiratory failure. We calculated the standard mean difference (SMD), odds ratio (OR), and their 95% confidence interval (95% CI) utilizing a fixed‐effect model or a random‐effect model according to the level of heterogeneity.

**Results:**

As of 14 May 2021, 13 articles were included in our meta‐analysis. The results showed that low albumin and uric acid levels were the risk factors for type II respiratory failure in acute exacerbation of COPD patients, and the differences were statistically significant (albumin: SMD = −2.03, 95% CI: −2.81, −1.26; uric acid: SMD = −1.28, 95% CI: −1.41, −1.15). Besides, 10 other systematic markers have been reported to be the risk factors for type II respiratory failure of patients with acute exacerbation of COPD, but only in single study.

**Conclusion:**

The meta‐analysis results further confirm that low albumin and uric acid levels are risk factors for type II respiratory failure in acute exacerbation of COPD patients. Additionally, this analysis also summarizes many emerging inflammatory indicators, nutritional indicator, and cardiovascular system indicators to predict the progression of acute exacerbation of COPD to type II respiratory failure but only in single study.

**Abbreviation**
AECOPD: Acute exacerbation of chronic obstructive pulmonary disease
ALB: Albumin
CI: Confidence interval
COPD: Chronic obstructive pulmonary disease
NLR: Neutrophil‐to‐lymphocyte ratio
NOS: Newcastle–Ottawa Scale
NT‐pro BNP: N‐terminal pro‐Brain Nitric Peptide
OR: Odds ratio
SD: Standard deviation
SMD: Standard mean difference
UA: Uric acid

## 1 INTRODUCTION

Chronic obstructive pulmonary disease (COPD) is a preventable and treatable disease, characterized by persistent respiratory symptoms and airflow limitation. In COPD patients, airflow limitation is usually progressive and even develops into type II respiratory failure in severe cases. This disease has a high mortality rate, seriously affecting people's physical health.^1^ However, the pathogenesis of COPD is not yet fully clarified. COPD has been reported to be associated with infection, exposure to cigarette smoke, inhalation of dust and harmful gases, and genetics.^2^ Additionally, chronic inflammation of the airway, lung parenchyma, and pulmonary vessels is a characteristic change in COPD, suggesting a pivotal role of inflammation and inflammatory cytokines in the pathogenesis and deterioration of COPD.^2^ Local airway inflammation leads to the enhancement of oxidative stress and apoptosis through the production of reactive oxygen species (ROS) and reactive nitrogen species (RNS) by inflammatory cells, such as neutrophils, macrophages, and cytotoxic T lymphocyte, thereby resulting in further deterioration of the condition.^3^, ^4^ Increased expression of various inflammatory cytokines, such as interleukin (IL)‐1, IL‐3, IL‐6, and tumor necrosis factor (TNF)‐ transforming growth factor (TGF), has been confirmed in diseases. For COPD, TNF‐IL‐1 and IL‐6 enhance the inflammatory process and contribute to some of the systemic effects of COPD.^2^

Acute exacerbation of COPD (AECOPD), as a critical is a process in the progression of COPD, is manifested as acute onset, deterioration of respiratory symptoms beyond daily variation, and ultimately leading to changes in drug treatment.^5^ AECOPD patients with further deterioration will have cardiopulmonary dysfunction, metabolic dysfunction, and finally type II respiratory failure.^6^ Type II respiratory failure is a complication contributing the most to the high mortality and poor prognosis of AECOPD.^6^ At present, the diagnosis of AECOPD mainly depends on the clinical manifestations. Specifically, the diagnosis is based on the sudden changes in patient's subjective feeling (baseline dyspnea, cough, and/or expectoration) beyond daily variation, which is difficult to judge.^7^ The importance of inflammation in the development of AECOPD has been proved, and the progression of AECOPD further increases the burden on the heart.^7^ The aim of this review therefore was to summarize the risk factors (mainly biomarkers) for type II respiratory failure in AECOPD patients, so as to guide timely clinical treatment and reduce the serious impact of COPD on human health.

## 2 METHODS

The systematic review followed the methodology outlined in Cochrane Handbook for Systematic Reviews of Interventions Version 6.0.^8^ And this study was reported based according to the PRISMA‐P (Preferred Reporting Items for Systematic Reviews and Meta‐Analyses Protocols).^9^

### 2.1 Search strategy

PubMed, Cochrane, Web of Science, WangFang Data, and the China National Knowledge Infrastructure were systematically searched for articles on risk factors of type II respiratory failure in AECOPD from the inception to 14 May 2021. The search items were as follows: “COPD,” “respiratory failure,” and “risk factor.” In addition, the references of the initially included articles were also in a systematic search for preventing omission and comprehensively reporting the risk factors of type II respiratory failure in AECOPD patients.

### 2.2 Inclusion and exclusion criteria

Based on the inclusion and exclusion criteria, two researchers independently evaluated the studies obtained from the initial search through the titles and abstracts. During this process, the disagreement was resolved through discussion. Inclusion criteria were as follows^1^: retrospective case–control studies^2^, studies with AECOPD patients as study subjects, the patients were divided into the study group (with type II respiratory failure), the control group (without type II respiratory failure),^3^ and studies with the following information of the AECOPD patients: age, course of disease, nosocomial infection or not, acid–base imbalance, other laboratory results such as blood uric acid (UA), albumin (ALB), and D‐dimer. Exclusion criteria were as follows^1^: duplicate publication of the same trial^2^ and incomplete data and relevant data unavailable from reasonable channels^3^, with major deficiencies in study design or major biases in reporting results. Studies that met any of the criteria were excluded.

### 2.3 Data extraction and quality assessment

Two researchers independently extracted the following data provided by the included literature: title, first author, publication year, number of study subjects, grouping, age, inclusion criteria and exclusion criteria of AECOPD patients, diagnostic criteria for AECOPD and respiratory failure, laboratory test results, results of the investigation, and related indicators of study design (mainly including study protocol and quality control).

On completion of data extraction, they checked the consistency of the extracted data. The quality of included case–control studies was assessed according to the Newcastle–Ottawa Scale (NOS). The evaluation items included^1^: appropriate selection of cases and controls or not: the definition, source, and comparability of cases and controls^2^ and appropriate determination of exposure or not. The included observational studies with scores of 6–9 were of high quality, 4 or 5 were of moderate quality, and 3 or less were of poor quality. If there was disagreement in the quality assessment, they settled it through discussion.

### 2.4 Statistical analysis

Results were merged across studies with STATA version 15.1 (Stata Corp MP., College Station, TX, USA).^10^, ^11^ Study subjects in each included study were AECOPD patients, and the control group was AECOPD cases without type II respiratory failure in the hospital, suggesting a good consistency. Assessment of heterogeneity was performed using *Q* test and *I* ^2^ statistics. *I* ^2^ values of 0%–39%, 40%–59%, and 60%–90% indicated low, moderate, and high heterogeneity among studies, respectively.^8^ In case of low heterogeneity, a random‐effect model was adopted for pooling results; otherwise, a fixed‐effect model was employed. For each binary variable, odds ratio (OR) and its 95% confidence interval (CI) were utilized to compare whether this variable was a risk factor for AECOPD with type II respiratory failure, while for each continuous variable, standard mean difference (SMD) and 95% CI were used. Additionally, pooled standard deviation (SD) of the studies was calculated by referring to Cochrane handbook.^8^ If the number of studies evaluating risk factors for type II respiratory failure in AECOPD patients was ≥5, the results were presented as forest plots; otherwise, the results were presented in tables. If the number of studies was ≥ 5, Egger's test was used for assessing the publication bias of the results and Duval and Tweedie's trim and fill test for the sensitivity of the results.^12^, ^13^ Exact *P* values would be reported unless *P* < 0.001. Except that *P* < 0.10 in the result of Egger's test was considered statistically significant, significant differences were suggested in other results if *P* < 0.05.

## 3 RESULTS

### 3.1 Literature search, study characteristics and quality assessment

Totally, 2221 and 13 articles were obtained by database retrieval and manual retrieval, respectively. On completion of exclusion of 673 duplicate articles, 1529 were then excluded through titles and abstracts (not related to chronic obstructive pulmonary disease, *n* = 621; review or in vitro/animal studies or letter or editorial or conference paper, *n* = 196; not related to type II respiratory failure caused by COPD, *n* = 243; not related risk factors, *n* = 469).

**FIGURE 1:**
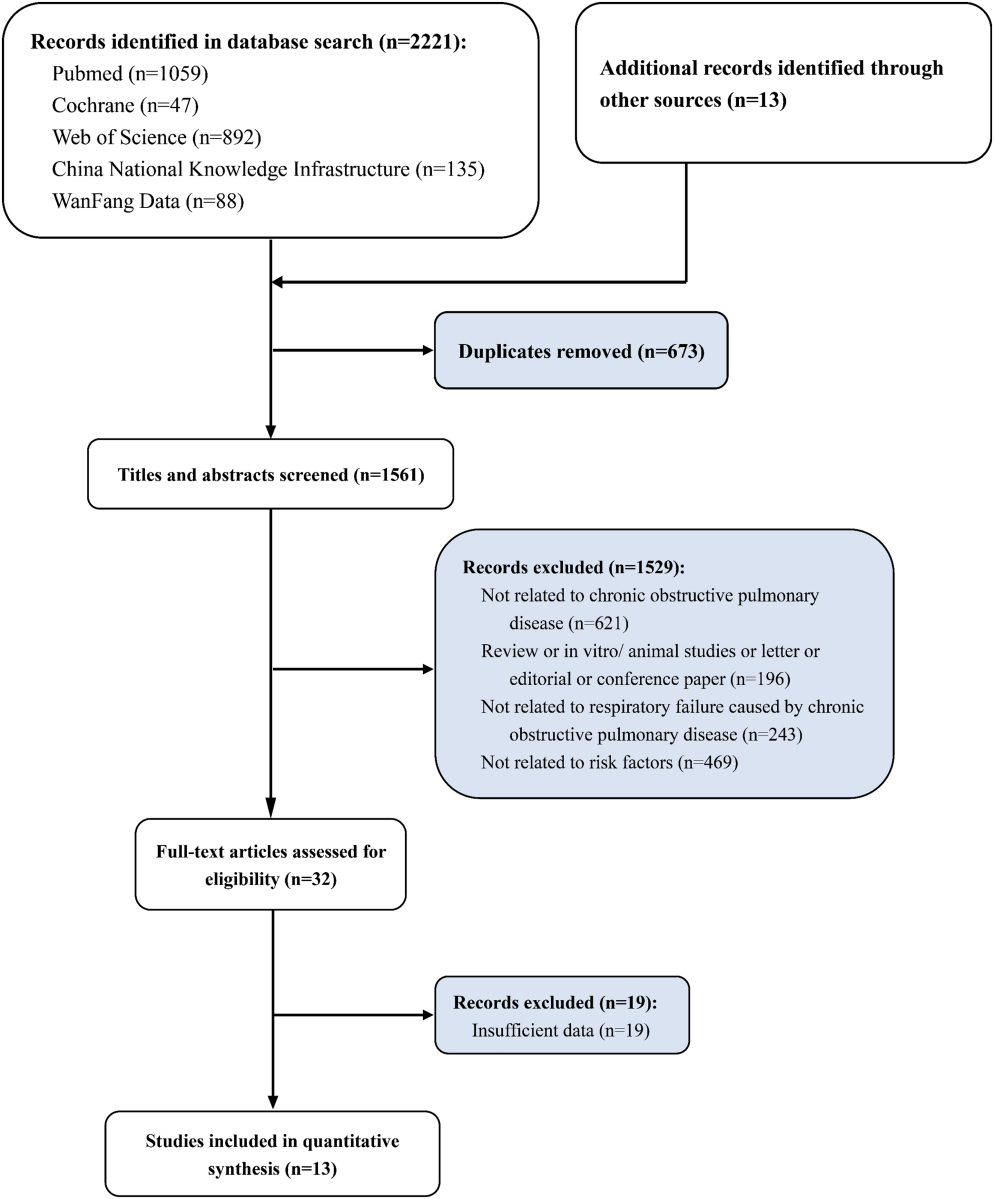
Study selection flowchart, a meta‐analysis of blood biomarkers associated with acute type II respiratory failure in chronic obstructive pulmonary disease (COPD)

The full texts of the remaining 32 studies were read for evaluation, and 19 of them were excluded because they could not provide valid data. Finally, 13 articles^14^, ^15^, ^16^, ^17^, ^18^, ^19^, ^20^, ^21^, ^22^, ^23^, ^24^, ^25^, ^26^ were included in this meta‐analysis (Figure 1), including 726 AECOPD patients with type II respiratory failure and 1418 AECOPD patients without type II respiratory failure. The basis characteristics of the 13 included studies are shown in Table 1.

**TABLE 1:** Baseline characteristics of included studies for meta‐analysis

Author, year	No. of cases		Age (years)	AECOPD diagnosis criteria	Res. failure definition	NOS score
	Res. failure	Con.				
Zhang (2020)^16^	48	69	74.04 ± 8.49	CECDT‐AECOPD	PaO_2_ < 60 mmHg and PaCO_2_ > 50 mmHg	6
Guo (2021)^19^	67	67	71.72 ± 3.05	CECDT‐AECOPD	PaO_2_ < 60 mmHg and PaCO_2_ > 50 mmHg	4
Chen et al. (2020)^17^	66	78	78.61 ± 6.51	CECDT‐AECOPD	PaO_2_ < 60 mmHg and PaCO_2_ > 50 mmHg	7
Bao (2016)^14^	48	58	76.28 ± 6.83	CECDT‐AECOPD	PaO_2_ < 60 mmHg and PaCO_2_ > 50 mmHg	7
Cheng et al. (2016)^15^	45	80	71.80 ± 5.60	CECDT‐AECOPD	PaO_2_ < 60 mmHg and PaCO_2_ > 50 mmHg	6
Liu (2014)^20^	52	96	75.18 ± 4.12	CECDT‐AECOPD	PaO_2_ < 60 mmHg and PaCO_2_ > 50 mmHg	5
Yu (2020)^21^	100	100	72.40 ± 3.50	CECDT‐AECOPD	PaO_2_ < 60 mmHg and PaCO_2_ > 50 mmHg	8
Liang et al. (2019)^22^	65	69	70.58 ± 6.34	CECDT‐AECOPD	PaO_2_ < 60 mmHg and PaCO_2_ > 50 mmHg	4
Song et al. (2019)^18^	32	468	59.60 ± 6.70	CECDT‐AECOPD	PaO_2_ < 60 mmHg and PaCO_2_ > 50 mmHg	8
Li (2014)^23^	48	88	74.83 ± 6.17	CECDT‐AECOPD	PaO_2_ < 60 mmHg and PaCO_2_ > 50 mmHg	5
Gu (2017)^24^	50	100	74.83 ± 5.50	CECDT‐AECOPD	PaO_2_ < 60 mmHg and PaCO_2_ > 50 mmHg	5
Chen (2017)^25^	45	85	74.70 ± 6.20	CECDT‐AECOPD	PaO_2_ < 60 mmHg and PaCO_2_ > 50 mmHg	6
Liu (2019)^26^	60	60	64.50 ± 11.40	CECDT‐AECOPD	PaO_2_ < 60 mmHg and PaCO_2_ > 50 mmHg	5

Abbreviations: CECDT‐AECOPD, Chinese expert consensus on the diagnosis and treatment of acute exacerbation of chronic obstructive pulmonary disease; Con., control; NOS, Newcastle–Ottawa Scale; Res., respiratory.

The results of the NOS scale‐based quality assessment of the 13 studies are presented in Table 1. All the studies had scores of 4–8, suggesting no studies with low quality. Before the analysis, patients with the following complications were excluded from each study^1^: combined with severe lesions of vital organs such as liver and kidney^2^; combined with malignant tumors, hematological diseases, and severe infections (such as respiratory system)^3^; and combined with mental disorder. Therefore, there was no significant data loss in each study, causing no significant damage to the power of the test but affecting the extrapolation of the study results. Collectively, the studies included in this meta‐analysis were of good quality, and the study results were of high reliability.

### 3.2 Comparison between patients with AECOPD accompanying type II respiratory failure and patients with AECOPD alone

**FIGURE 2:**
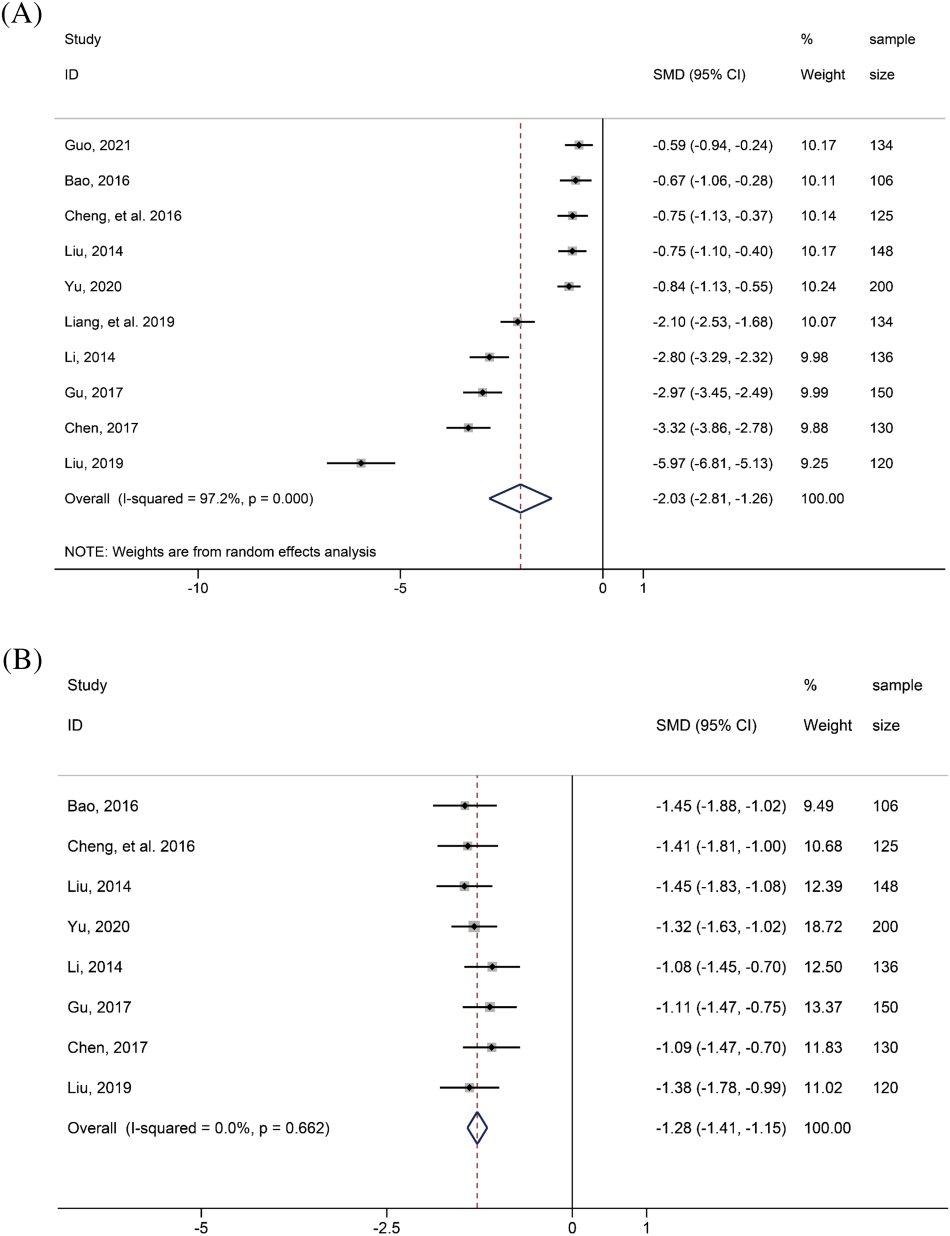
Forest plot of comparison between patients with acute exacerbation of chronic obstructive pulmonary disease (AECOPD) accompanying type II respiratory failure and patients with AECOPD alone: (A) albumin; (B) uric acid

**ALB.** Ten studies reported ALB. A random‐effect model was adopted for determining whether ALB was a risk factors for type II respiratory failure in AECOPD patients. The results showed that AECOPD patients with type II respiratory failure had lower ALB level than those without type II respiratory failure, and the difference was statistically significant (SMD = −2.03, 95% CI: −2.81, −1.26) (Table 2 and Figure 2A). Notably, the heterogeneity of this indicator was as high as 97.2%, and the significance of this result was required further discussion.

**TABLE 2:** Summarized results of included studies

Indicators	No. of studies	Sample size	Effect size (95%CI)	Heterogeneity (%)	
				*I* ^2^	*P*
**Comparison between patients with AECOPD accompanying respiratory failure and patients with AECOPD alone**					
ALB	10	1383	−2.03 (−2.81, −1.26)	97.2	<0.001
UA	8	1115	−1.28 (−1.41, −1.15)	0.0	0.662
Acid–base imbalance	3	400	18.02 (10.76, 30.20)	0.0	0.982
Nosocomial infection	4	607	12.09 (7.34, 19.91)	0.0	0.759
D‐dimer	1	144	0.48 (0.15, 0.81)	‐	‐
NLR	1	117	0.94 (0.55, 1.32)	‐	‐
NT‐pro BNP	1	117	1.05 (0.66, 1.44)	‐	‐
HCY	1	144	0.58 (0.24, 0.91)	‐	‐
VEGF < 135 g/L	1	500	4.39 (1.86, 10.34)	‐	‐
CRE > 133 μmol/L	1	500	3.21 (1.41, 7.30)	‐	‐
hs‐CRP > 17.5 mg/L	1	500	4.53 (2.09, 9.80)	‐	‐
CRP	1	134	1.72 (1.32, 2.12)	‐	‐
IL‐8	1	134	1.20 (0.83, 1.57)	‐	‐
TNF‐α	1	134	1.19 (0.83, 1.56)	‐	‐

*Note*: VEGF < 135 g/L, CRE > 133 μmol/L, hs‐CRP > 17.5 mg/L; effect size is OR; for the other indicators, effect size is SMD. For indicators (acid–base imbalance; nosocomial infection.

Abbreviations: AECOPD, acute exacerbation of chronic obstructive pulmonary disease; ALB, albumin; CRE, creatinine; CRP, C‐reactive protein; HCY, homocysteine; hs‐CRP, high‐sensitivity C‐reactive protein; IL‐8, interleukin‐8; NLR, neutrophil‐to‐lymphocyte ratio; NT‐pro BNP, N‐terminal pro‐brain nitric peptide; TNF‐α, tumor necrosis factor‐α; UA, uric acid; VEGF, vascular endothelial growth factor.

UA. Eight studies reported UA. A fixed‐effect model was utilized and found that AECOPD patients with type II respiratory failure had lower UA level than those without type II respiratory failure, and the difference was statistically significant (SMD = −1.28, 95% CI: −1.41, −1.15) (Table 2 and Figure 2B).

Three studies reported acid–base imbalance. The meta‐analysis result revealed that the proportion of acid–base imbalance in AECOPD patients with type II respiratory failure was significantly higher than that in patients with AECOPD alone (OR = 18.02, 95% CI: 10.76, 30.20) (Table 2). Four studies reported nosocomial infection. The meta‐analysis showed that the proportion of nosocomial infection in AECOPD patients with type II respiratory failure was markedly higher than that of patients with AECOPD alone (OR = 12.09, 95% CI: 7.34, 19.91) (Table 2).

In addition to the above factors, there are 10 biomarkers have been reported might be risk factors of AECOPD patients combine with type II respiratory failure but only in single study (Table 2). They may contribute to future research of AECOPD combined with type II respiratory failure.

### 3.3 Publication bias assessment and sensitivity analysis

We used Egger's test to analyze the publication bias of the indicators. The test results found no publication bias in both ALB and UA. Further, Duval and Tweedie's trim and fill test revealed that the effect sizes of these two indicators were stable and instructive (Table 3).

**TABLE 3:** Evaluation of publication bias and sensitivity analysis

Index	Egger's regression		Duval and Tweedie's trim and fill		
	Intercept	*p*	Original effect size	Studies trimmed	Adjusted effect size
ALB	−3.083	0.415	−2.03 (−2.80, −1.26)	0	−2.03 (−2.80, −1.26)
UA	−1.618	0.631	−1.28 (−1.41, −1.14)	1	−1.31 (−1.43, −1.18)

Abbreviations: ALB, albumin; UA, uric acid.

## 4 DISCUSSION

COPD is a common and frequently occurring disease in respiratory medicine, which has a high incidence in the elderly. COPD, with long course of the disease, is progressive and poses a great threat to the physical and mental health of patients.^27^ Acute respiratory failure is a common complication of COPD, contributing the most to the poor prognosis.^27^ Analysis of the risk factors for acute respiratory failure caused by COPD and subsequently corresponding intervention are of high value in improving the prognosis of patients.^15^

The results of meta‐analysis in each study showed that the decrease of ALB and UA levels was a risk factor for type II respiratory failure in AECOPD patients, highlighting the necessity of monitoring these two indicators during treatment and taking corresponding means for intervention. Although the meta‐analysis results of ALB showed a high heterogeneity, all studies reported ALB pointed to the same conclusion that the decrease of ALB level was a risk factor for type II respiratory failure in AECOPD patients. However, due to the high heterogeneity, we cannot conclude when ALB decreases to what level we need to be alert to the occurrence of type II respiratory failure. The main reason for dyspnea in some patients whose condition has been effectively controlled lies in the fact that the relationship between airway resistance and respiratory muscle load is mainly positively correlated. That means that when the airway resistance is increased, the respiratory muscle load will also increase, thus causing an increase of the respiratory muscle oxygen consumption and consequently dyspnea.^28^ If this condition persists for a long time, it will accelerate the patient's energy consumption and metabolism at rest, leading to inadequate amounts of the calories, nutritional imbalance, and severe weight loss.^27^, ^29^ Both body mass index and ALB level can reflect the energy reserve of the body, but we believe that ALB has a higher accuracy. To sum up, we believed that ALB might be the critical risk factor for type II respiratory failure in AECOPD patients as a metabolic consequence. Therefore, COPD patients should improve their nutritional status, especially for those with hypoproteinemia. Besides, we suggest that the low level of UA in AECOPD patients complicated with type II respiratory failure may be related to the progression of body inflammation in AECOPD patients. UA has the effect of scavenging oxygen free radicals and contribute to alleviate the oxidative stress of the patients.^30^ With the aggravation of COPD, oxides increase while UA level decreases in the body,^30^ suggesting the significance of monitoring UA levels and taking appropriate interventions during the treatment of COPD to prevent further aggravation.

COPD patients are in a state of long‐term chronic hypoxia, and therefore, anaerobic glycolysis of cells predisposes to the production of acidic products and consequently acidosis. When complicated with acute type II respiratory failure, the anaerobic glycolysis of cells and the CO_2_ retention state are prone to result in electrolyte disturbance and acid–base imbalance, further aggravating the condition.^29^ Respiratory tract infection is a crucial cause of AECOPD, which is often clinically treated with broad‐spectrum antibiotics and inhaled glucocorticoids. However, treated patients have decreased immune function and are prone to dysbacteriosis, resulting in nosocomial infection and increasing the risk of acute type II respiratory failure.^29^

AECOPD is associated with chronic inflammation, primarily affecting the lung parenchyma and surrounding respiratory tract and subsequently resulting in irreversible progressive airflow limitation. This kind of inflammation is characterized by increased numbers of alveolar macrophages, neutrophils, T lymphocytes, and innate lymphoid cells.^31^ As an emerging inflammatory marker, neutrophil‐to‐lymphocyte ratio (NLR) is a parameter derived from complete blood counts and has been applied to assess AECOPD.^31^ This review also summarized that AECOPD patients with type II respiratory failure had significantly higher NLR levels than those without type II respiratory failure. With progression to type II respiratory failure, AECOPD patients have increased cardiac load and even occur symptoms such as heart failure.^32^ The production of N‐terminal pro‐brain nitric peptide (NT‐pro BNP), which directly related to ventricular volume expansion and ventricular pressure overload, is one of the effective indicators of congestive heart failure.^32^ For example, Zhang et al. has proved that NT‐pro BNP is an independent risk factor for death in AECOPD patients with type II respiratory failure, and detection of NT‐pro BNP is helpful for determining the condition.^16^ D‐dimer is a cross‐linked fibrin degradation product and thrombotic marker commonly used in the diagnosis of pulmonary embolism and venous thromboembolism.^17^ Hypoxia promotes disorders of blood coagulation and fibrinolysis, and elevated D‐dimer levels are associated with activation of fibrinolytic system and thrombosis.^17^ Chen et al. have revealed that the D‐dimer level before treatment is higher in the type II respiratory failure group than in the group without type II respiratory failure.^17^ In addition to the above mentioned, factors associated with systemic inflammation, such as homocysteine, vascular endothelial growth factor, high‐sensitivity C‐reactive protein, C‐reactive protein, IL‐8, and tumor necrosis factor‐α had been reported positively associated with an increased risk of type II respiratory failure in patients with AECOPD. And serum creatinine which is a muscle metabolite of the body increased in our body might indicate that the body and muscle consumption and metabolism increase. It could reflect the nutritional status of the organism as an auxiliary factor and indicate the recovery of respiratory muscle function being affected.^18^

This study still has some limitations. First, the studies included in this review are mainly Chinese ones, and more studies from all over the world are required to compare the results and to expand the scope of influence of the meta‐analysis results. Second, many risk factors have been only reported in a single literature, and more relevant studies on these factors are required for determining their value in clinical practice.

In conclusion, the meta‐analysis of this review further confirms that low ALB and UA levels are risk factors for type II respiratory failure in AECOPD patients. Additionally, this analysis also summarizes many emerging inflammatory indicators, nutritional indicator, and cardiovascular system indicators to predict the progression of AECOPD to type II respiratory failure but only in single study.

## ETHICS STATEMENT

Ethical approval was not needed because this is a meta‐analysis.

## CONSENT FOR PUBLICATION

Not applicable.

## CONFLICT OF INTEREST

All the authors declare that they have no conflict of interest.

## AUTHOR CONTRIBUTION

STY and FL: critical revision of the manuscript; STY and FL: substantial contribution to the conception and design of the work and manuscript drafting; STY and FL: acquisition, analysis, and interpretation of the data; STY and FL: revising the manuscript critically and final approval of the version to be published. All authors have read and approved the final manuscript.

## FUNDING INFORMATION

None.

## AUTHOR CONTRIBUTIONS

Tieying Shi, Li Feng: Critical revision of the manuscript; Tieying Shi, Li Feng: Substantial contribution to the conception and design of the work, manuscript drafting;Tieying Shi, Li Feng: Acquisition, analysis, and interpretation of the data;Tieying Shi, Li Feng: Revising the manuscript critically, final approval of the version to be published.All authors have read and approved the final manuscript.

## Data Availability

The datasets used and/or analyzed during the current study are available from the corresponding author on reasonable request.
